# Illuminating the Co‐Phosphoregulatory Network of the Dark Kinase STK10 by Omics Approaches

**DOI:** 10.1155/bmri/7315694

**Published:** 2026-10-07

**Authors:** Vaishnavi Gopalakrishnan, Amal Fahma, Althaf Mahin, Leona Dcunha, Athira Perunelly Gopalakrishnan, Samseera Ummar, Rajesh Raju, Sowmya Soman

**Affiliations:** ^1^ Centre for Integrative Omics Data Science (CIODS) Yenepoya (Deemed to be University) Mangalore Karnataka India

**Keywords:** co-regulation, phosphoproteomics, predominant phosphosites, STK10

## Abstract

Serine/threonine‐protein kinase 10 (STK10), an ERM protein kinase, functions as a membrane–cytoskeleton linker and regulates lymphocyte cortical rigidity, cell shape, adhesion, and migration. Despite its emerging relevance in immune regulation and cancer, the phosphoregulatory mechanisms governing STK10 remain poorly understood. To address this, we systematically assembled and mapped the global phosphoproteomic datasets on the dark STK10 phosphosites. From 453 quantitative differential profiles representing STK10 Class 1 phosphosites, 12 predominant sites were identified based on frequency. Of these, four phosphosites (T185, S191, S514, and T952) were conserved with the homologous kinase SLK, indicating evolutionary conservation. Notably, S450, S455, T14, S448, and S454 were predicted as potential autophosphorylation sites, suggesting intrinsic regulatory control. Predictions indicated that PAK2 and MAP3K2 may act as both upstream kinases and downstream substrates of STK10, with their phosphosites (MAP3K2_S153 and PAK2_S141) consistently co‐regulated, suggesting a common regulatory axis. These findings stacked the phosphorylation landscape of STK10, extending regulatory insights beyond its kinase domain. This phosphoproteomics data‐driven study establishes a framework for prioritizing functional validation and structural investigations to define the physiological and pathological significance of dark phosphosites in STK10 in immunity and cancer.

## 1. Introduction

STK10, a member of the STE20‐like protein family, is a serine/threonine kinase implicated in diverse cellular processes such as cytoskeletal organization, cell migration, cell adhesion, and cell cycle progression [1]. STK10, also known as lymphocyte‐orientated kinase (LOK), is predominantly expressed in lymphoid tissues such as spleen and thymus [2]. It is also found highly expressed in hematopoietic cells [1]. Structurally, it comprises an N‐terminal kinase domain and a C‐terminal coiled‐coil region [3] and possesses significant sequence homology with human SLK and *Xenopus laevis* xPlkk1, the known polo‐like kinase kinases [1].

Although some insights into the biological functions of STK10 are perceived, it still remains a dark kinase, as its regulatory as well as mechanistic roles are largely unknown. It is mainly involved in lymphocyte migration through phosphorylation of ERM (ezrin, radixin, and moesin) proteins, which regulate cell shape and motility [4, 5]. STK10 is also linked to leukocyte adhesion and invasion of immune cells and immune checkpoint molecules [6, 7]. Its suppression is associated with the activation of immunogenic cell death (ICD) and alteration of antitumor response [8]. STK10 is known to phosphorylate PLK1, through which it influences cell cycle progression [1]. A recent study by Li et al. demonstrated the regulatory role of STK10 in platelet function by interacting with integrin‐linked protein kinase (ILK). STK10 knockout mice produced impaired hemostasis and reduced susceptibility to arterial thrombosis, implying its therapeutic potential against cardiovascular diseases [9].

Emerging evidence envisages the context‐dependent paradoxical role of STK10 in tumorigenesis. Its overexpression has been associated with acute myeloid leukemia [6], uveal melanoma [10], lung cancer [4], and Ewing′s sarcoma [11]. Knockout of STK10 inhibits cell migration in prostate cancer; however, it promotes migration in cervical cancer [12, 13]. Hence, its role as an oncogene or tumor suppressor could be context‐dependent. STK10 is also shown to influence the tumor microenvironment by recruiting cytotoxic T lymphocytes and modulating angiogenesis, perceiving its potential as an immunotherapy target [12, 13]. Structural studies indicate that targeting its uniquely extended back pocket enables the development of highly selective STK10 inhibitors [14].

Despite growing recognition of its biological significance and paradoxical behavior in cancers, the regulation and downstream signaling events associated with STK10 remain largely uncharacterized. Structural studies hint that its activation segment dimerization (residues 189–193) promotes autophosphorylation of nonconsensus sites, offering a possible regulatory mechanism [15]. Furthermore, STK10 is considered as a basophilic kinase with an unusual preference for tyrosine at the P‐2 position in its substrate peptides [5]. Currently, 43 phosphosites in STK10 are reported in PhosphoSitePlus, but their functional roles and upstream regulatory kinases are still unresolved [16]. As a key factor in modulating kinase activity and also their substrate recognition, exploring the role of major phosphosites in STK10 is critical, especially upon consideration of its context‐dependent functions. The experimental approaches to explore their role are impeding and demand novel approaches to filter potentially relevant phosphoproteins for targeted analysis. This study integrates large‐scale phosphoproteomics datasets to generate insights into the phosphorylation‐directed regulatory mechanisms and signaling network of STK10 through the analysis of its prominently co‐regulated phosphoproteins.

## 2. Materials and Methods

### 2.1. Compilation of STK10 Global Phosphoproteomics Datasets

The global datasets obtained using the search terms “phosphoproteomics OR phosphoproteome, NOT plant, NOT review” at the level of human cells and tissues were screened for the detection of STK10 phosphosites. Subsequently, datasets containing Class 1 phosphosites of STK10 were selected based on the localization probability of ≥ 75% or an A‐score of ≥ 13. These Class 1 phosphosite‐reported datasets were then divided into qualitative profile datasets (where test and control are considered as independent phosphosite profile datasets) and quantitative differential datasets (where test biological or experimental conditions are compared with their corresponding control phosphosite datasets). The quantitative differential datasets were subsequently categorized based on respective statistical thresholds with a fold change cutoff (≥ 1.3, upregulation; ≤ 0.76, downregulation) and *p*‐value (< 0.05). Each protein and phosphosite was mapped to its corresponding gene symbols and UniProt accession numbers using in‐house scripts.

### 2.2. Distribution of Detected STK10 Phosphosites Across Phosphoproteomes

To determine the frequency of detection of STK10 phosphosites across datasets, we analyzed the compiled data containing the Class 1 phosphosites in both qualitative profile datasets and quantitative differential datasets. The most frequently detected phosphosites across multiple profiling and differential datasets were categorized as predominantly detected phosphosites.

Multiple sequence alignment was performed to check the conservation of predominant phosphosites using Clustal Omega. Further, the co‐occurrence of differentially expressed phosphosites under the same experimental condition in the assembled quantitative datasets was analyzed [17]. The frequency of reported differential co‐regulation of the phosphosite pairs is provided in Table S1.

### 2.3. Phosphoproteins Often Co‐Differentially Regulated With STK10 Phosphosites

To identify the phosphosites in other proteins (PsOPs) that display either a positive or negative co‐regulation with the predominant phosphosites in STK10, we separately compiled the quantitative differential datasets. When the expression of the predominant phosphosite is upregulated and the expression of PsOPs is also upregulated, it is labeled as UU. Likewise, if the expression of the predominant phosphosite and PsOPs was downregulated, it is considered DD. Collectively, both UU and DD indicated positive co‐regulation (UUDD). On the other hand, when the expression of predominant phosphosite is upregulated and the expression of PsOPs is downregulated, it is labeled as UD, and vice versa, it is considered DU. Both UD and DU indicated negative co‐regulation (UDDU). We next conducted a one‐sided Fisher′s exact test (FET) derived from a contingency table [18, 19]. The following criteria were applied to filter high‐confidence PsOPs that co‐differentially regulated with each of the predominant STK10 phosphosites. Firstly, the *p*‐value from FET cutoff was set below 0.05. There should be at least three distinct research articles and three distinct experimental conditions supporting unidirectional (either positive, else negative) co‐regulation. The co‐regulation ratio *Σ*(*n*UU + *n*DD)/*Σ*(*n*UD + *n*DU) or *Σ*(*n*UD + *n*DU)/*Σ*(*n*UU + *n*DD) should account for at least 15% frequency of predominant phosphosites.

### 2.4. Identification of Phosphosites in Kinases and Interactors Co‐Regulated With STK10 Sites

Experimentally validated upstream kinases associated with STK10 phosphosites were sourced from PhosphoSitePlus [16], Phospho.ELM Version 9.0 [20], and RegPhos 2.0 [21]. Predicted upstream kinases were sourced from NetworKIN [22] and AKID [23], and additionally from iKiP‐DB [24], which is derived from a high‐throughput in vitro screen of 385 kinases. Both kinases and substrates (above 90% threshold), cataloged in the study by Johnson et al. [25], were extracted. The experimentally validated interactors of STK10 were extracted from HPRD [26], BIND [27], BioGRID [28], ConsensusPathDB Release 35 [29], CORUM [30], and RegPhos 2.0 [21].

### 2.5. Kinase–Substrate Enrichment Analysis (KSEA)

Kinase–substrate analysis was carried out using the KSEA app [31]. Positively co‐regulated (UUDD) PsOPs that passed significance filters (*p* < 0.05 and fold change ≥ 2) were used as input. The kinase–substrate search was restricted to the PhosphoSitePlus database, ensuring that only experimentally validated interactions were included. The tool was specifically used to identify upstream kinases corresponding to the input phosphosites, without inferring differential kinase activity.

### 2.6. Data Visualization and Gene Ontology (GO) Enrichment Analysis

For data visualization, RAWGraphs 2.0 (10.1145/3125571.3125585) and Cytoscape [32] were used to represent relationships among the proteins. R/Bioconductor package trackViewer (10.18129/B9.bioc.trackViewer) is used for generating lollipop plots. GO analysis was performed using Enrichr to identify enriched biological processes, molecular functions, and cellular components associated with the co‐regulated proteins [33]. The family relationships between the STK10, MAP3K2, and PAK2 kinases were represented using the KinMap tool to analyze their distribution among different kinase families [34].

## 3. Results

### 3.1. Human Cellular Phosphoproteomes With STK10 Phosphosite Regulation

We screened over 3825 publicly available articles in PubMed for gathering global human cellular phosphoproteomics datasets of STK10. From those datasets, we obtained 1072 qualitative profiling data with the detection of STK10 Class 1 phosphosites and 453 datasets with differential regulation across diverse conditions. Through systematic mapping, 35 phosphosites in the profiling datasets (Figure 1A) (Table S2) and 27 phosphosites in the quantitative datasets were identified as differentially regulated across diverse experimental conditions (Figure 1B) (Table S3). Phosphosites across these qualitative and quantitative differential datasets, along with details about their experimental conditions, are available in Table S4. Notably, the phosphosites S264, T256, T390, and S387 in the profile datasets were not reported in PhosphoSitePlus. Among the differentially regulated phosphosites, four phosphosites (T185, S191, T195, and T259) are located in the kinase domain, whereas other sites are located outside the domain [35].

**Figure 1:**
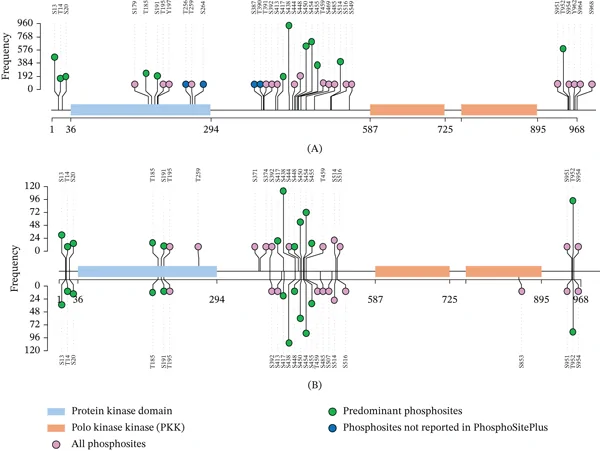
Lollipop plot visualization of the frequency of the STK10 phosphosites. Each node represents the phosphosites. Class 1 phosphosites in STK10 across the currently assembled datasets. (A) Detected in 1072 global cellular qualitative profiles. (B) Regulated in 453 global quantitative differential profile datasets.

Considering the frequency of detection and the diverse experimental conditions in which they were regulated, 12 phosphosites were identified as predominant phosphosites of STK10 (Figure 2A) (Table S5), as their recurrent phosphorylation is indicative of potential functional relevance [36]. Because STK10 exhibits elevated kinase activity in pancreatic ductal adenocarcinoma (PDAC) [37], cProSite (Cancer Proteogenomic Data Analysis Site)–based phosphosite expression analysis of STK10 further revealed that several predominant phosphosites are dysregulated across multiple cancer types (Figure 2B) (Table S6) [38].

**Figure 2:**
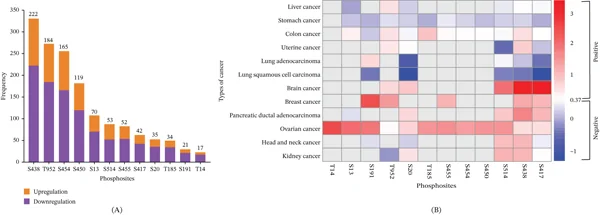
STK10 phosphosite frequency and cancer‐specific regulation. (A) STK10 phosphosites with their differential expression across quantitative differential datasets. The *x*‐axis shows individual STK10 phosphosites, and the *y*‐axis shows their frequency (the total number of datasets in which each site was detected to be regulated). (B) Heatmap showing the log fold change expression of STK10 phosphosites across different cancer types based on the cProSite data. Higher and lower phosphorylation levels reflect the differences observed between tumor and corresponding normal tissues.

### 3.2. Sequence Conservation of STK10 Predominant Phosphosites With SLK

Conservation of phosphosites between homologous kinases often indicates regulatory hotspots [39]. To explore this, predominant phosphosites of STK10 were compared with those of SLK, a closely related member of the GCK‐V (germinal center kinase group V) subfamily of the STE20‐like serine/threonine kinase family [1]. Four sites of STK10 (T185, S191, S514, and T952) were found to be considerably conserved (Figure 3A). Notably, S191 and T185 of STK10 correspond to S189 and T183 of SLK (STE20‐like serine/threonine‐protein kinase), which are known autophosphorylation sites associated with SLK′s kinase activation [40]. Given their localization within the kinase domain (residues 36–294), S191 and T185 are inferred to engage similar roles in STK10 kinase activity.

**Figure 3:**
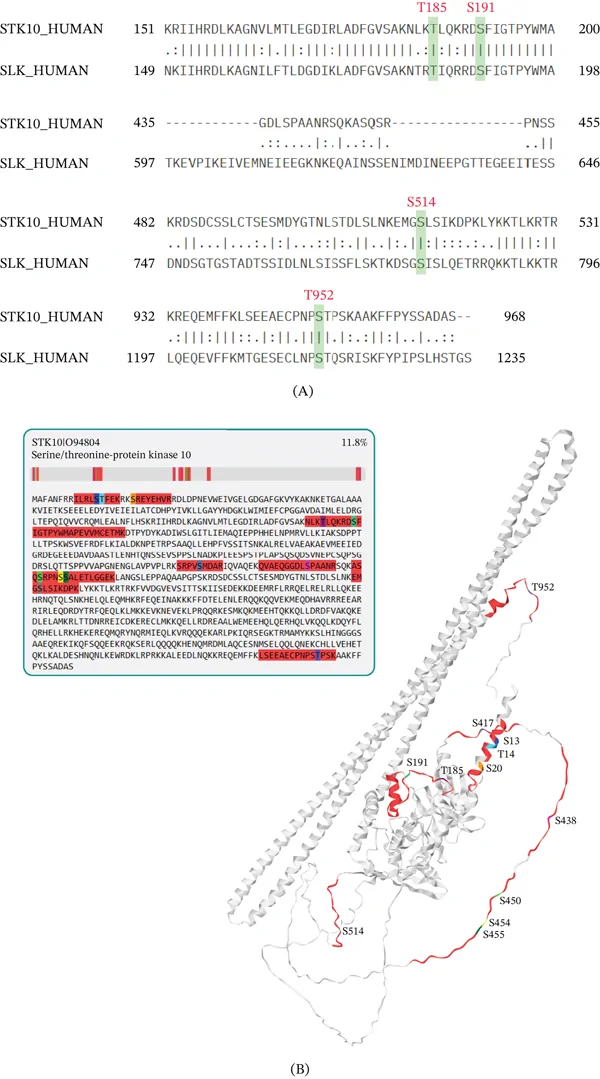
(A) Conservation of STK10 phosphosites with SLK. (B) The predicted 3D structure of the STK10 (serine/threonine‐protein kinase 10) kinase generated by AlphaFold, highlighting the predominant phosphosites in different colors to visualize the locations of these phosphosites within the protein. Areas depicted in red indicate the peptide coverage linked to these sites. The sequence view inset in panel B aligns with the structural representation, marking peptide coverage in red and phosphosites with corresponding color highlights.

### 3.3. Analysis of Potential Co‐Regulation Pattern Between STK10 Phosphosites

Phosphorylation sites that are detected together, especially those reported co‐differentially regulated frequently or located in close sequence or structural proximity, are likely to be functionally related and may be regulated by the same kinases [41]. To explore such patterns, a co‐occurrence analysis was performed [17]. Notably, S455 showed positive co‐regulation with S20, whereas T185 showed positive co‐occurrence with S514 (Figure 4). S455 and S20 were identified to be positively co‐regulated in 14 differential datasets while negatively co‐regulated in one dataset. T185 and S514 were identified to be positively co‐regulated in 12 differential datasets and negatively co‐regulated in one dataset. A negative co‐regulation was observed between S951 and T185, T259 and S20, S444 and S417, S954 and T185, and S954 and S516 (Table S1). This provides insights to explore potential co‐functional roles and, further, their structural organization toward specific phosphoregulated functions by designing targeted experiments in the future. All the predominant phosphosites mapped in the predicted AlphaFold structure are given in Figure 3B.

**Figure 4:**
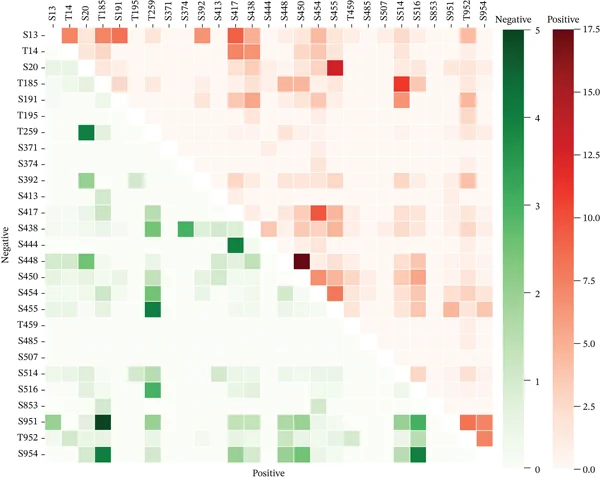
Heatmap representing positive and negative co‐occurrence patterns of phosphosites within STK10. Positive co‐occurrence with frequency of positive co‐regulation over negative co‐regulation ≥ 3 is represented in the upper red triangle, and negative co‐occurrence with frequency of negative co‐regulation over positive co‐regulation ≥ 3 is represented in the lower green triangle. Co‐occurrence frequencies below three are represented with a gray shade.

### 3.4. Proteins and Their Phosphosites Frequently Co‐Regulated With STK10 Phosphosites

To explore the potential functional role of STK10 predominant phosphosites, co‐regulating phosphorylation events in other proteins were analyzed. Because phosphorylation events that occur together can regulate specific biological processes, a list of co‐regulated phosphorylated proteins was compiled. These co‐regulated phosphoproteins were categorized into positively co‐regulated (UUDD) and negatively co‐regulated (UDDU) proteins (Table S7). Specific criteria were employed to identify high‐confidence co‐regulated phosphoproteins. Three predominant STK10 phosphosites, such as S438, T952, and S454, showed positive co‐regulation with 10, 36, and 14 phosphoproteins, respectively. There were no negatively co‐regulated phosphoproteins after applying the criteria for these sites. Further, predominant sites of STK10 have the following number of co‐regulated proteins: S450 (UUDD: 31 and UDDU: 1), S13 (290 and 10), S514 (368 and 12), S455 (335 and 59), S417 (371 and 92), S20 (610 and 285), S191 (1092 and 83), T185 (445 and 11), and T14 (429 and 102). The top high‐confidence expression‐co‐regulated PsOPs of predominant STK10 sites are provided in Figure 5. Because of the limited number of positively co‐regulated phosphoproteins identified for S438, T952, S454, and S450 after stringent filtering, these sites were excluded from comparative GO visualization. For the remaining predominant phosphosites, the top 100 positively co‐regulated proteins ranked by co‐regulation frequency were used to ensure uniform input size for comparative enrichment analysis across phosphosites. To prioritize the most statistically significant enrichments, only the top three biological processes with the lowest adjusted *p*‐values for each phosphosite were given in Figure 6 (Table S8).

**Figure 5:**
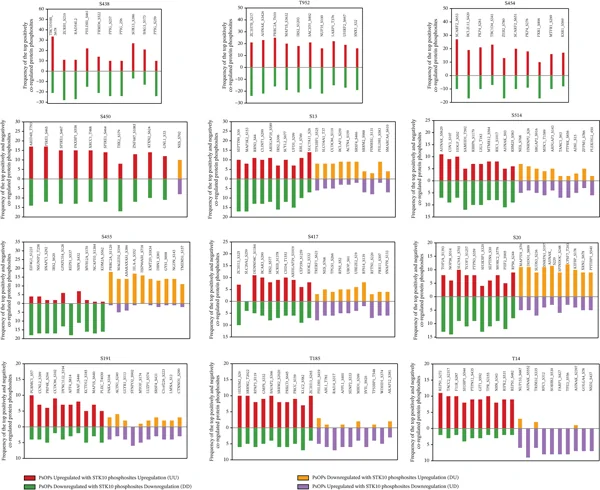
Top high‐confidence positively and negatively co‐regulated PsOPs of predominant STK10 phosphosites.

**Figure 6:**
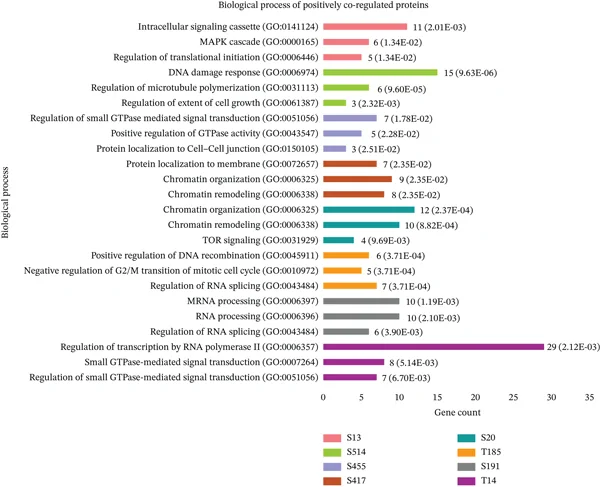
Biological processes enriched in positively co‐regulated proteins of STK10 predominant phosphosites. The *x*‐axis represents the number of genes involved in each biological process, and the *y*‐axis represents the corresponding biological process terms. The values in brackets represent the −log_10_(adjusted *p*‐value), indicating the statistical significance of enrichment. Color‐coded bars indicate corresponding phosphosites (S13, S514, S455, S417, S20, T185, S191, and T14).

### 3.5. Potential Phosphosite‐Centric Regulation of STK10 Binary Interaction

Considering the regulatory role of phosphorylation in modulating protein–protein interactions, the interaction network of STK10 in co‐regulated phosphoproteins was examined. From different databases, a total of 49 binary interactors and two STK10 protein complexes were assembled, comprising 260 proteins. The positive co‐regulation of phosphosites in these binary interactors with STK10 predominant phosphosites is given in Table S9. The network representing high‐confidence co‐regulated binary interactors of STK10 is given in Figure 7.

**Figure 7:**
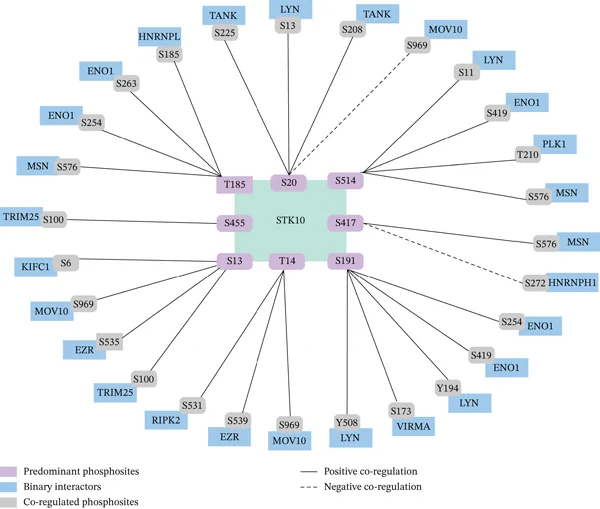
A network representing high‐confidence co‐regulated binary interactors of STK10.

Among them, EZR, MSN, and PLK1 are experimentally validated downstream substrates of STK10. EZR phosphorylation at S535 and S539, both located in the C‐terminal F‐actin binding domain, was found to be co‐regulated with S13 and T14 phosphosites in STK10. Similarly, MSN_S576, also situated in the C‐terminal F‐actin binding domain, was co‐regulated with STK10 sites S417, S514, and T185. PLK1 enzymatic activity‐inducing site T210 in its kinase domain co‐regulated with S514 of STK10 [42]. PLK1_T210 is currently associated with DNA repair and cell cycle regulation [43, 44].

### 3.6. Co‐Regulation Analysis of Predicted Upstream Kinases of STK10 Phosphosites

To elucidate the upstream regulatory mechanisms governing STK10 phosphorylation, particularly at these predominant phosphosites, kinase prediction analysis was performed. The candidate kinases were identified based on the study by Johnson et al. [25] and using prediction tools such as NetworKIN/AKID and iKiP‐DB (Table S10). The co‐regulation of these potential upstream kinase phosphosites with STK10 phosphosites is represented in Figure 8. Notably, NetworKIN/AKID predicted PRKCD as the upstream kinase for S13, T185, and S191, sites of STK10. Johnson et al. [25] categorized AKT1 as the upstream kinase for S13, T14, S191, and S417.

**Figure 8:**
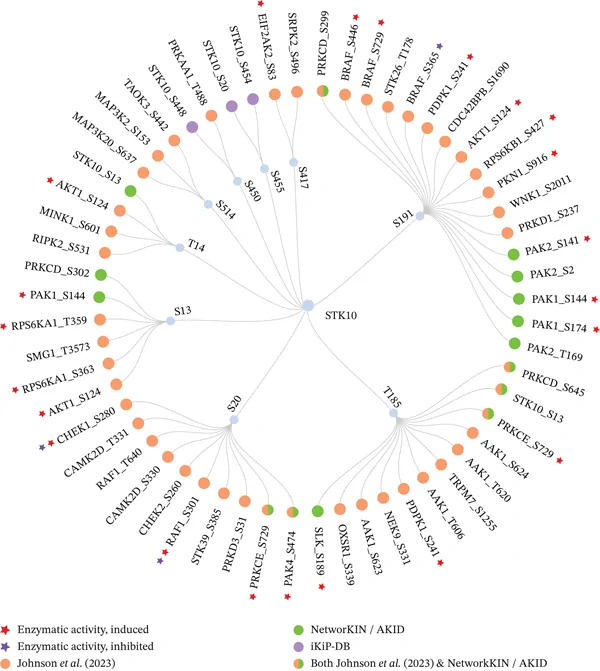
Circular dendrogram representing potential upstream kinases of STK10 predominant phosphosites and phosphosites in upstream kinases that are co‐regulated with STK10 predominant phosphosites.

### 3.7. Extraction of the Potential Substrates of STK10 Based on Phosphosite‐Centric Co‐Regulation Analysis

Recent findings suggest broader functional roles of STK10, highlighting the potential existence of additional, yet uncharacterized, STK10 substrates that demand systematic investigation. Johnson et al. [25] have categorized 4367 proteins as substrates for STK10. Further, iKiP‐DB listed some proteins as substrates of STK10. Many of these predicted substrates harbored phosphosites positively co‐regulated with STK10 predominant phosphosites (Figure 9) (Table S11). Specifically, STK10_S191 was positively co‐regulated with 83 PsOPs, which are predicted substrates. Other predominant phosphosites, including S417, S20, T185, T14, S13, S455, S514, T952, S450, and S454, were positively co‐regulated with phosphosites in 56, 50, 44, 39, 38, 36, 32, 3, 2, and 2 predicted substrates, respectively.

**Figure 9:**
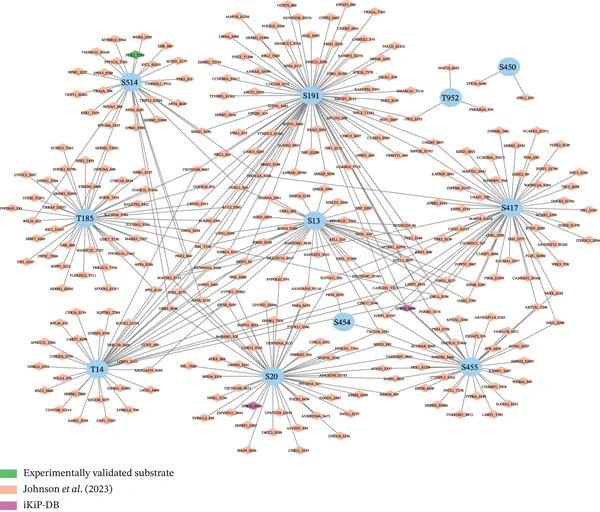
Network representing potential substrates of STK10 and phosphosites in substrates that are positively co‐regulated with predominant phosphosites of STK10.

These predicted substrates co‐regulated with the 12 predominant phosphosites were largely unique, with only limited overlaps. Each site displayed distinct co‐regulated phosphoproteins and predicted substrates. Notably, some predicted substrates, including MAP3K2_S153, TBC1D4_S341, EML3_S156, ZZEF1_S1537, SLC7A11_S26, DENND4C_S1184, UFD1_S299, RB1_T356, PAK2_S64, STK10_S454, LARP1_S824, and TBC1D10B_S687, were positively co‐regulated with different predominant sites of STK10, representing the small subset of common overlaps.

### 3.8. Autophosphorylation Potential of STK10

STK10 has been reported to exhibit autophosphorylation activity; however, the sites that are regulated by autophosphorylation have not yet been experimentally verified. We identified predominant STK10 phosphosites that were positively co‐regulated with other STK10 phosphosites. Apart from the co‐regulation of the site pairs mostly in the same phosphopeptides, such as S450:S448, T14:S13, and S455:S454, we also obtained phosphosite pairs such as S455:S20 in distinct peptides. For these predominant phosphosites (S450, S455, and T14), STK10 was predicted as an upstream kinase (Figure 8). Many of the co‐regulated phosphosites (S448, S454, and S455) of STK10 were also predicted as downstream substrates (Figure 9). This included co‐regulation between STK10 sites within the same peptides, such as S450:S448 and S455:S454, and between sites in distinct peptides, such as S417:S454, S20:S455, and S191:S454. These patterns suggest that the phosphosites S450, S455, T14, S448, and S454 may represent potential autophosphorylation sites and that STK10 could be a kinase with context‐dependent self‐regulation potential.

### 3.9. KSEA and Functional Concordance of Potential Substrates With STK10 Kinase Domain Phosphosites

To identify upstream kinases potentially responsible for regulating the positively co‐regulated phosphosites (PsOPs), we performed KSEA, restricted to experimentally validated interactions curated in the PhosphoSitePlus database (Table S12). To highlight kinases with substantial regulatory representation, only those with ≥ 10 substrates in the KSEA dataset were considered. These included 27 CDK2 substrates at S191, 17 CDK1 substrates at S191, 14 CSNK2A1 substrates at S20, 12 CDK2 substrates at S20, and 10 CDK2 substrates at T14. Notably, CDK2 and CDK1 substrates were enriched in the positively co‐regulated proteins of eight (S13, S191, S20, S455, S514, T14, T185, and S417) and six (S191, S20, S455, S514, T14, and T185) predominant phosphosites. But the regulatory site CDK2_Y15 was positively co‐regulated with only two predominant phosphosites, T185 and S514. Y15 resides within the kinase domain, and its dephosphorylation is essential for CDK2 activation and cell cycle progression [45].

Further, kinase‐dead mutants of STK10 have been shown to cause significant aberrations in cell cycle progression [1]. To determine whether phosphorylation sites within the kinase domain (T185 and S191) may contribute to this regulation, we analyzed their co‐regulated proteins. At the protein level, T185 was co‐regulated with 372 proteins, of which 92 were annotated as cell cycle regulators, whereas S191 was co‐regulated with 776 proteins, including 194 cell cycle–related proteins. At the phosphosite level, S191 showed co‐regulation with 1092 phosphosites across 776 proteins, among which 45 sites from 38 proteins were associated with cell cycle regulation. Similarly, T185 was co‐regulated with 445 phosphosites across 372 proteins, including 10 sites from nine proteins linked to cell cycle processes. Taken together, these observations align with existing literature on the role of STK10 in cell cycle, underscoring the robustness and reliability of our data.

## 4. Discussion

Our systematic phosphoproteomic study of STK10 identified 12 predominant phosphosites with potential functional significance. Two sites, S191 and T185, were located in the kinase domain; S13, T14, and S20 were located near the N‐terminal; S417, S438, S450, S454, and S455 were positioned adjacent to the regulatory domain spanning residues 332–422 [46, 47]; T952 was situated within the C‐terminal region. Among these, four phosphosites (T185, S191, S514, and T952) were found to be conserved with its closely related kinase SLK. Notably, phosphoproteomic studies in PDAC identified STK10 as an active kinase associated with aggressive tumor behavior and poor prognosis [37]. Consistently, data from the cProSite, a web‐based platform for visualizing proteomic and phosphoproteomic data from multiple tumor types, revealed that several predominant phosphosites of STK10, particularly S438, S417, and S514, showed moderate upregulation across cancers, with significant upregulation observed in brain cancer [38]. Collectively, it emphasizes the potential involvement of these phosphosites in dynamic regulatory functions.

Analysis of co‐regulation patterns between predicted upstream kinases and substrates revealed several potential autophosphorylation sites, including S450, S455, T14, S448, and S454. The region that promotes dimerization (amino acids 332–422) aids in the transautophosphorylation of residues located in the activation loop of the kinase domain [15]. Interestingly, two kinases, such as PAK2 and MAP3K2, were predicted as both upstream kinases and downstream substrates of STK10 and often co‐regulated with STK10. Kinome phylogenetic tree analysis using KinMap showed the distribution of STK10, MAP3K2, and PAK2 into the STE family of kinases (Figure S1). Based on our datasets, STK10_S191 positively co‐regulated with PAK2_S141, an activity‐inducing site as well as an autophosphorylation site in PAK2 [48, 49]. With STK10_S191 phosphomotif being a consensus PAK2 substrate recognition motif (Figure S2), PAK2 is likely to phosphorylate STK10 at S191. Furthermore, as PAK2_S141 was also predicted as a substrate of STK10 and as S191 is present in the kinase domain of STK10 (phosphomotif of PAK2_S141 partially matches the STK10 substrate preference motif [Figure S4]), we also propose a potential feedback loop between STK10 and PAK2.

Similarly, in the case of MAP3K2, STK10_S514 is co‐regulated with MAP3K2_S153. This phosphosite S153 stabilizes MAP3K2 and inhibits its ubiquitination [50]. Both were predicted to phosphorylate each other by Johnson et al. [25]. Further, STK10_S514 also carries a consensus MAP3K2 substrate recognition motif (Figure S3). This predominant phosphosite S514 may be an activity‐associated site of STK10, as it is co‐regulated with PLK1_T210, which is a well‐known experimentally validated substrate of STK10, and T210 is the activation site of PLK1 [42]. Similarly, the phosphomotif of MAP3K2_S153 also harbors a partial consensus STK10 substrate preference motif (Figure S4) and is consistent with the reported preference of STK10 for substrates containing nearby basic residues [5]. Hence, STK10 may also phosphorylate MAP3K2 at S153.

A study by Li et al. [9] revealed that PKC could modulate STK10 phosphorylation. In line with this, our analysis showed that predicted PKCs (PRKCD and PRKCE) as upstream kinases were co‐regulated with STK10 kinase domain phosphosite T185. Another phosphosite, S191, in the kinase domain, was co‐regulated with PRKCD, which was also predicted as an upstream kinase for this same site.

Co‐regulation analysis of predicted substrates by Johnson et al. [25] suggests that besides ERM proteins, STK10 likely phosphorylates a wide range of substrates, consistent with the recent study that identified ILK as a novel substrate of STK10 in platelets. Further, KSEA showed that substrates of CDK1 and CDK2 were enriched in positive co‐regulated proteins of predominant sites.

Functional contributions of predominant site phosphorylation remain speculative and could also be attributed to the absence of robust structural data. Through the phosphosite‐centric approach undertaken in this study, we filtered the kinases, substrates, and interactors of STK10 that also aligned with existing literature on functional attributes of STK10. It underscores the robustness and reliability of our approach for their selection for targeted experimental validation. More significantly, our approach entails and helps to visualize the phosphosite‐level co‐regulation between proteins, either as kinases, substrates, or interactors. Future studies integrating structural analysis and experimental data are crucial for establishing the physiological relevance of STK10‐mediated phosphoregulation toward phosphosite‐directed therapeutics for immune and neurological disorders and cancers.

## 5. Limitations

Although this study was performed based on the bioinformatics analysis of phosphoproteomics datasets available from PubMed‐indexed articles, certain limitations should be acknowledged. The assembled datasets were compiled from multiple independent experiments conducted under various conditions and analytical workflows. Different cell types, mass spectrometry platforms, phosphopeptide enrichment strategies, and quantitative approaches may contribute to dataset heterogeneity. Additionally, the findings observed in this study are largely based on co‐regulation patterns of phosphoproteins, which require future experimental validation. Despite these limitations, this work provides an overview of STK10 phosphoregulation and aids site‐directed investigations in future research.

## 6. Conclusion

This study presents a phosphoproteomic profiling of STK10, highlighting predominant phosphorylation sites and their regulatory interconnections. Our results show the significance of phosphosites outside the kinase domain, and co‐regulated predicted substrates provide the broad regulatory function of STK10. By integrating phosphosite‐centric regulation patterns to predicted upstream kinases, substrates, and interactors, we provide the first phosphoregulatory network of STK10 as a valuable resource for future experimental evaluation of its potential paradoxical regulation and its role as a signaling nexus in pathophysiological processes.

## Author Contributions

Vaishnavi Gopalakrishnan: Formal analysis, investigation, visualization, and writing original draft. Amal Fahma: Formal analysis and review and editing. Althaf Mahin: Formal analysis, writing, and review and editing. Leona Dcunha: Review and editing. Athira Perunelly Gopalakrishnan: Data curation. Samseera Ummar: Software. Rajesh Raju: Conceptualization, project administration, resources, and supervision and review. Sowmya Soman: Resources, methodology, review and editing, and supervision and review.

## Funding

No funding was received for this manuscript.

## Ethics Statement

The authors have nothing to report.

## Conflicts of Interest

The authors declare no conflicts of interest.

## Supporting Information

Additional supporting information can be found online in the Supporting Information section.

## Supporting information


**Supporting Information 1** Table S1: Co‐occurrence data of STK10 phosphosites.

**Supporting Information 2** Table S2: Figure 1A. Frequency of the STK10 phosphosites in global cellular qualitative profiles datasets.

**Supporting Information 3** Table S3: Figure 1B. Frequency, upregulation, and downregulation of STK10 phosphosites in global quantitative differential phosphoproteomic datasets.

**Supporting Information 4** Table S4: Table 1A. Human cellular profile data. Table 2A. Human cellular differential data.

**Supporting Information 5** Table S5: Figure 2A. STK10 predominant phosphosites with their differential expression across quantitative differential datasets.

**Supporting Information 6** Table S6: Figure 2B. The log fold change expression of STK10 predominant phosphosites across different cancer types based on the cProSite data.

**Supporting Information 7** Table S7: High‐confidence PsOPs co‐regulated with STK10 expression (after applying filtering parameters).

**Supporting Information 8** Table S8: Gene ontology of positively co‐regulated proteins of STK10 predominant phosphosites.

**Supporting Information 9** Table S9: Co‐regulated PsOPs in binary interactors of STK10.

**Supporting Information 10** Table S10: Co‐regulated PsOPs in upstream kinases of STK10.

**Supporting Information 11** Table S11: Co‐regulated PsOPs in downstream substrates of STK10.

**Supporting Information 12** Table S12: Kinase–substrate enrichment analysis (KSEA) result of co‐regulated PsOPs in STK10.

**Supporting Information 13** Figure S1: Kinase phylogenetic tree generated using KinMap, highlighting STK10, MAP3K2, and PAK2. The highlighted kinases illustrate their distribution across distinct kinase families. Figure S2: Sequence logo representing the substrate preference motif of PAK2 generated using experimentally validated PAK2 substrates obtained from PhosphoSitePlus. Figure S3: Sequence logo representing the substrate preference motif of MAP3K2 generated using experimentally validated MAP3K2 substrates obtained from PhosphoSitePlus. Figure S4: Sequence logo representing the substrate preference motif of STK10 generated using experimentally validated STK10 substrates obtained from PhosphoSitePlus.

## Data Availability

The data that support the findings of this study are available in the Supporting Information of this article.
