# Supplementary material for: Illuminating the Co‐Phosphoregulatory Network of the Dark Kinase STK10 by Omics Approaches
Source: Biomed Res Int. 2026 Oct 7;2026:7315694. doi: 10.1155/bmri/7315694 (PMC13643830; doi:10.1155/bmri/7315694)
Supplement: Supplementary file 1 [file BMRI-2026-7315694-s009.docx]

**Title:** **Illuminating the co-phosphoregulatory network of the dark kinase STK10 by omics approaches**

I. **Kinase Family Distribution of STK10, MAP3K2, and PAK2**

**Supplementary Figure S1.** Kinase phylogenetic tree generated using KinMap, highlighting STK10, MAP3K2, and PAK2. The highlighted kinases illustrate their distribution across distinct kinase families.

II. **PAK2 substrate preference motif**

**Supplementary Figure S2.** Sequence logo representing the substrate preference motif of PAK2 generated using experimentally validated PAK2 substrates obtained from PhosphoSitePlus.

**Phosphomotif**

| **Protein** | **Phosphosite** | **Phosphomotif Sequence** |
| --- | --- | --- |
| STK10 | S191 | ktLQKRDsFIGtPyW |

III. **MAP3K2 substrate preference motif**

**Supplementary Figure S3.** Sequence logo representing the substrate preference motif of MAP3K2 generated using experimentally validated MAP3K2 substrates obtained from PhosphoSitePlus.

**Phosphomotif**

| **Protein** | **Phosphosite** | **Phosphomotif Sequence** |
| --- | --- | --- |
| STK10 | S514 | SLNKEMGsLSIKDPK |

IV. **STK10 substrate preference motif**

**Supplementary Figure S4.** Sequence logo representing the substrate preference motif of STK10 generated using experimentally validated STK10 substrates obtained from PhosphoSitePlus.

**Phosphomotif**

| **Protein** | **Phosphosite** | **Phosphomotif Sequence** |
| --- | --- | --- |
| PAK2 | Ser141 | tVkQKyLsFtPPEkd |
| MAP3K2 | Ser153 | AERKKRLsIIGPTSR |
